# Economic evaluation of implementation strategies in health care

**DOI:** 10.1186/s13012-014-0168-y

**Published:** 2014-12-18

**Authors:** Ties Hoomans, Johan L Severens

**Affiliations:** Institute of Health Policy & Management, Erasmus University Rotterdam, Rotterdam, The Netherlands; Section of Hospital Medicine, Department of Medicine, University of Chicago, Chicago, USA; Institute of Medical Technology Assessment (iMTA), Erasmus University Rotterdam, Rotterdam, The Netherlands

**Keywords:** Implementation strategies, Cost of implementation, Economic evaluation, Decision-making

## Abstract

Economic evaluations can inform decisions about the efficiency and allocation of resources to implementation strategies—strategies explicitly designed to inform care providers and patients about the best available research evidence and to enhance its use in their practices. These strategies are increasingly popular in health care, especially in light of growing concerns about quality of care and limits on resources. But such concerns have hardly motivated health authorities and other decision-makers to spend on some form of economic evaluation in their assessments of implementation strategies. This editorial addresses the importance of economic evaluation in the context of implementation science—particularly, how these analyses can be most efficiently incorporated into decision-making processes about implementation strategies.

## Editorial

### Introduction

Economic evaluation assesses the efficiency and allocation of resources to interventions that may improve health care and health outcomes. Economic evaluation applies not only to decisions about interventions or services that directly target patients, like pharmacological treatments and medical devices, but also to decisions about implementation strategies, which are explicitly designed to inform care providers and patients about the best available research evidence and to enhance its use in their practices.

Many inefficiencies in health-care delivery result from overuse of unnecessary services, underuse of beneficial interventions, or medical errors [1]. In light of the growing concerns about the quality of care and budgetary pressures, implementation strategies are used to improve service delivery and outcomes. Potentially effective strategies that can promote an uptake of services can be as straightforward as clinical decision support, education and financial incentives—or can be as complex as total quality management and reforms of health-care systems.

Empirical studies of the effects of implementation strategies related to behavior change and health outcomes have become more numerous [2,3]. Many explore how to most effectively address particular problems of implementation, a question that may be answered with insights into the mechanisms by which implementation works and the use of behavior change theory, from disciplines such as psychology and sociology [4,5].

But although the prominence of implementation science in health services is increasing, relatively little attention has been paid to another important aspect of implementation strategies: these efforts demand resources, and thus, have costs. Depending on the perspective of the decision-maker and their objective(s), the cost of implementation may include the following: 1) costs associated with executing implementation strategies; 2) the excess cost of service delivery as uptake or implementation changes; 3) the opportunity cost to providers and patients partaking in the implementation activities; and 4) research and development-related expenses resulting from the process of implementing change in health care. Unless the budget for implementation is sufficient, not all possible implementation projects can be supported. Trade-offs must be made, and these trade-offs merit an analysis that can compare costs to their benefits and that can identify the opportunity cost of choices—in other words, an economic evaluation.

**Figure 1:**
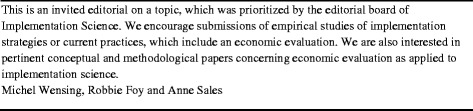
**Editors’ note.**

This editorial addresses the importance of economic evaluation in the context of implementation science—in particular, how these analyses can be most efficiently incorporated into decision-making processes about implementation strategies (Figure 1).

### Use of economic evaluation

Despite the prevalence of economic evaluation in health services research, its use is not standard practice in assessing implementation strategies. Recent reviews revealed fewer than 60 studies of the efficiency of strategies for implementing clinical practice guidance before 2008 [2,6,7], with no substantial progress since then.^a^ The number of economic evaluations contrasts sharply with the number of studies on implementation strategies assessing only their effect on behavior change and health outcomes.

Why are implementation decisions so seldom guided by economic evaluation? Some of the more plausible reasons include divergent views on cost and cost-effectiveness, limited resources for evaluative research, and the paucity of data for decision-making.

Views on the appropriate role of economics in evaluating implementation strategies may differ importantly between people and over time. Some revert to basic ethical tenets and moral obligations to discard information on cost completely; others view, more realistically, considerations of cost as secondary or complementary to other criteria, such as clinical effectiveness. But regardless of the differences of opinion and how they impact evaluation and decision-making, particular implementation strategies do have costs. Simply ignoring these implications can have undesirable consequences, such as inefficiencies and inequities that compromise the accessibility and delivery of health services—the very reason why spending on implementation of research evidence is considered initially.

Limits on research capacity and appropriate data seem plausible reasons for decision-makers not to base implementation decisions on some form of economic evaluation, but in fact conceal a paradox: implementation decisions need economic evaluations that produce good-quality data for these decisions to be well-informed; economic evaluations need decisions that utilize their results for these evaluations to be supported.

### Methods of economic evaluation

Methods—cost/cost-consequences analysis, cost-effectiveness/utility analysis, or cost-benefit analysis—do not need to pose a burden to performing economic evaluation of implementation strategies. The approach is similar to the economic evaluation of the services being implemented, while explicitly accounting for the resources used in developing and executing implementation strategies as a cost of ensuring appropriate service delivery [8,9]. Determining which method to use requires weighing potential uses in resource-allocation decision-making versus its demand of information and computational complexity (Table 1).

**Table 1:** **Overview of forms of economic evaluation**

**Form of evaluation**	**Use for decision making**	**Measurement of health effects**	**Economic summary measure**
Cost-consequences analysis	Comparison of implementation strategies that have disparate outcomes	Any measure	Not applicable
Cost-effectiveness analysis	Comparison of implementation strategies that produce a common outcome	Process measures (e.g., professional guidance adherence, patient compliance to medication) or health effects (intermediate or final), measured in natural units	Cost-effectiveness ratio (e.g., cost per case averted, cost per life-year saved), at patient or population level
Cost-utility analysis	Comparison of implementation strategies that have morbidity and mortality outcomes	Final health outcomes, including health status, patient preferences, utilities	Cost per quality-adjusted life-year, at patient or population level
Cost-benefit analysis	Comparison of implementation strategies with different units of outcome (health and nonhealth)	Monetary units	Net health benefit or net monetary benefit, at patient or population level
Cost analysis	Comparison of net cost of implementation strategies with equivalent outcomes	Not applicable	Net cost or cost of illness, at patient or population level

#### Cost and cost-consequences analysis

Common to all forms of economic evaluation is the analysis of cost. Properly conducted—collecting adequate data on the use of all relevant resources on implementation and assigning appropriate tariffs or prices to those resources—cost analysis can help decision-makers address a not unimportant question: How much more will it cost to pursue implementation efforts? (Depending on the question and the purpose of the cost information, costing may require detailed analysis and such accuracy-focused methodology as micro or activity-based costing.) But information from cost analysis, such as budget impact analyses or patient level cost-minimization studies, generally is insufficient to determine whether intervening in implementation problem(s) makes economic sense. Unless the potential alternative strategies are certain to have the same health outcomes across provider and patient practices over time—which is unlikely—implementation decisions will require a joint comparison of costs and outcomes by full economic evaluation.

At the simplest level, economic evaluation entails the mere listing of all cost/benefit implications of each potential choice, as in cost-consequences analyses [10]. This form of economic evaluation was applied in a trial-based study of task substitution for diagnosing fibromyalgia in inpatients [11]. Compared to a specialist-led process, a nurse-led process was reported to have higher patient satisfaction scores, equivalent health outcomes, and lower consumption of care and other resources.

Analyses like these have distinct uses. They provide information for spending decisions to address problems in health care, when possible implementation strategies are expected to have outcomes that are too disparate to be combined meaningfully. Cost-consequences analyses permit value judgments without having to fully specify a relation between all the different measures of outcomes. And yet listing the cost/benefit implications of implementation strategies alone fails an important objective of economic evaluation—to make explicit the opportunity costs of alternative resource uses.

#### Cost-effectiveness and cost-utility analyses

These opportunity costs can be assessed directly using other forms of economic evaluation: cost-effectiveness or cost-utility analyses. Incremental cost-effectiveness ratios are established by dividing the difference in costs of various implementation strategies by the corresponding difference in health outcomes. Again, the measure(s) of outcomes most appropriate for ratio calculations depends, to an important extent, on the objective of decision-making and the perspective of analysis. Common metrics typically used include incremental cost per life-year gain or per quality-adjusted life-year (QALY).

Ratios of cost-effectiveness—absolute and relative—can vary considerably across targeted providers, patients, behaviors, practices, and services. A sample of cost-effectiveness studies and calculated incremental cost-effectiveness ratios are found in Table 2. These results indicate that actively promoting and implementing clinical guidance may provide an inefficient use of resources, yield life-years or QALYs at additional cost, or may even be cost-saving.

**Table 2:** **Examples of incremental cost-effectiveness ratios and suggested decisions about implementation strategies**

**Study**	**Comparison of implementation strategies**	**Intervention considered for implementation**	**Incremental cost-effectiveness ratio**	**Suggestions for implementation decision**
Mason et al. 2005 [12]	Specialist-nurse led clinics versus usual care	Lipid control in patients with diabetes versus no lipid control	$19,950 per quality-adjusted life-year	Use of specialist-nurse led clinics for implementing lipid control is cost-effective
Scheeres et al. 2008 [13]	Multifaceted strategy, including health professional and patient education and instruction, versus usual care	Cognitive behavior therapy of chronic fatigue syndrome versus regular counseling	€5,320 per recovered patient	Use of multifaceted strategy for implementing cognitive behavior therapy is cost-effective
Walker et al. 2009 [14]	Financial incentives to primary care practices versus usual care	Use of ACE inhibitor and other quality indicators versus conventional care	£5,623 per quality-adjusted life-year	Use of financial incentives for implementing ACE inhibitor and other quality indicators is cost-effective
Hoomans et al. 2009 [15]	Audit and feedback to primary care physicians versus usual care	Intensive control of blood glucose in patients with type 2 diabetes versus conventional control	€25,640 per quality-adjusted life-year	Use of audit and feedback for implementing intensified control of blood glucose is cost-effective
Choudhry et al. 2011 [16]	No co-payments for patients versus co-payments	Preventive medication after myocardial infarction versus no preventive medication	$54 per nonfatal vascular event or vascularization averted (cost-saving)	Use of no co-payments for implementing preventive medication is cost-effective
Mortimer et al. 2013 [17]	Multifaceted strategy targeting primary care physicians, including interactive workshops, versus guideline dissemination alone	Evidence-based care for acute low back pain versus convention	−AU$108 per x-ray referral avoided (cost-saving)	Use of multifaceted strategy for implementing evidence-based care is cost-effective
Gillespie et al. 2014 [18]	Structured patient education with group follow-up versus individual follow-up	Self-management in type 1 diabetes versus conventional care	€19,300 per quality-adjusted life year (cost-saving)	Use of structured patient education with group for implementing self-management is not cost-effective

Wide variation in outcomes is not uncommon given the many types of information inputs used in cost-effectiveness studies. One obvious and important determinant is the cost of implementation; economies of scale and scope may apply as implementation strategies target larger groups of providers and patients, and multiple behaviors and practices. But the cost-effectiveness of such strategies also depends critically on the effect they have on provider and patient behaviors—as measured by guideline adherence and patient compliance—and on the differential outcomes of care between services being implemented. The greater the difference in expected outcomes between usual care and the change being implemented, and the more widespread the implementation, the more likely a strategy is to be cost-effective.

Translating cost-effectiveness ratios into resource allocation decisions can be difficult—even when potential implementation projects are comparable in scale and scope, and information about all the analysis inputs is so precise that the outcomes can be regarded as certain. Strategies that improve health and lower costs should be accepted; rejected should be those that worsen outcomes at higher costs. But what if a strategy is expected to improve (worsen) health outcomes but also cost more (less)?

In such cases, health or safety gains from implementation strategies need to be valued in monetary units, reflecting the budget constraints and opportunity cost of alternative resource uses. Common thresholds for choices of pharmacological treatments and other health services range from €20,000 to €80,000 per life-year or QALY—and similar threshold values may well apply to accept-reject decisions in an implementation context. Yet the critical question is: Do current thresholds fully incorporate the cost of implementation of ‘cost-effective’ health services?

#### Cost-benefit analysis

When assigned appropriate thresholds, cost-effectiveness data can be transformed to a more comprehensive measure of implementation strategy efficiency—the net benefit. As earlier applications of this concept suggested [14,15], the barriers to using so-called cost-benefit analysis (information-intensive, computationally complex) may often be overcome by the analytic benefits it has to offer: i) direct comparison of implementation projects of varying scale and scope, and ii) detailed assessment of uncertainty in implementation decision outcomes.

### Toward efficient use of economic evaluation

Once the methods for evaluation (including cost-effectiveness thresholds) are agreed upon, economic evaluation becomes a useful tool in the studying and planning of strategies for implementing change in health care. The question then becomes as follows: How can economic evaluation be performed most efficiently?

#### Limited collection of economic data

Economic evaluations are more efficient if data collection on outcomes is limited. One approach is to confine the study to the measures of the care process, say cost per change in professional guidance adherence or patient compliance to medication, instead of measuring actual health outcomes. For example, in a Dutch quasi-experiment on the use of financial incentives as an implementation strategy [19], incentivizing primary care providers was found to reduce prescriptions of targeted drugs, saving costs in comparison to usual care. Because there was also good evidence that denying patient medication did not have (long-term) effects on health outcomes, the incentive plan was likely to be a cost-effective strategy to implement more conservative prescribing practices in primary care.

Other ways to improve efficiency by limiting data collection include shortening the length of patient or provider follow-up or by relying on studies of less rigorous design for data collection.

However, limiting data collection can have undesirable consequences, such as reducing confidence in the accuracy of the conclusions drawn from the analysis. Consider the Dutch study of financial incentives [19]: can the established cost-effectiveness ratios be considered the same across all targeted care providers and prescription drugs? Or do these ratios actually vary by provider, drug, baseline prescriptions rate, or by some other source of heterogeneity? Has enough information been collected to ascertain whether incentivizing providers’ prescribing practices will have spillover effects to non-targeted behaviors and practices? Limiting the collection of economic data can increase evaluative efficiency, but the potential biases in the assessment of strategies need be carefully considered.

#### Use of decision analytic models

Practical considerations suggest yet another approach to economic evaluation and efficiency improvement: the use of decision analytic models. In modeling studies, economic data on implementation strategies may be synthesized from a range of sources, including theory on behavioral change, rather than from a single trial or observational study. For example, in a Dutch study of the implementation of intensified glycemic control in type 2 diabetes, the comparison of audit and feedback to primary care providers versus usual care was based on an economic model [15]. The model permitted establishing estimates of incremental cost per QALY ratios by linking behavior change to health-related outcomes using a simulation of experiments.

Decision analytic models have many more uses along these lines—for example, they can provide estimates of the expected value of information to form a basis for deciding whether additional data collection is necessary [20,21]. Despite apparent benefits, the use of models in economic evaluation and implementation choices remains uncommon.

#### Early assessment of implementation decisions

The greatest gain in efficiency for implementation decisions may not be the method of economic evaluation but rather the timing. Economic evaluations are typically being performed ex-post—after some inefficiency has been identified as a problem of implementation and after designing and testing a set of strategies.

Instead of carrying out economic evaluations after the fact, a 3-step ex-ante process of evaluation and decision-making is potentially much more efficient:

- Step 1: Assess the expected returns (as measured by net benefit on a monetary or health scale) from promoting the implementation of research evidence or any further change in clinical management or health policy through the use of value of implementation analysis [15,20,21].
- Step 2: Make predictions of the implementation cost, which may include research and development-related expenses and the opportunity cost of care providers and patients partaking in the implementation activities.
- Step 3: Set [1] against [2] before pursuing a more elaborate process of evaluation and decision-making regarding implementation strategies.

Early economic assessment can help eliminate cost-ineffective implementation studies early on, allowing resources to be directed toward problems in care where intervening is likely to be more beneficial. Knowledge of the practicality of health service implementation is essential. If valid and meaningful estimates of resource allocation requirements for implementation can be made, the cost of implementation could be considered much sooner in the health-care decision-making process—namely, when the decisions about care components are being made [22].

## Conclusions

Performing economic evaluations is not rocket science. Already commonly used in the assessment of health services, economic evaluation is a potentially useful tool for making decisions on strategies for implementing research knowledge into clinical practice, management, or health policy. Confronted with a problem of implementation, decision-makers who wish to derive the greatest benefits from available resources must spend on some form of economic evaluation. Only by comparing each potential choice across both costs and benefits can the opportunity cost of implementation strategies be assessed. As more economic evaluations are performed and decision-makers leverage analytic techniques such as modeling and value of implementation analysis, the contribution of economic evaluation to decision-making processes will increase. The question is *how* economic evaluation can most efficiently be incorporated into implementation decisions, not whether it should.

## Endnote

^a^A comprehensive search in PubMed for English language publications from 1 January 2008 to 1 July 2014, using the terms (in all fields): ‘economic eval* OR cost-effect*’, ‘implementation strateg* OR quality improv*’, revealed 284 records, most of which recorded publications of studies that did not report any cost of implementation or that refrained to perform an economic evaluation of the reported implementation or quality improvement strategy in terms of cost of implementation.
